# 

**Published:** 1879-03-20

**Authors:** 


### TABLE OF CONTENTS.

ORIGINAL AND SELECTED ARTICLES.

Typhoid Pneumonia—Its Treatment, &c., by Alban S. Payne, M.D., 81. Childbirth without Pain, by R. B. Anderson, M.D., 86. Chloral Hydrate in Obstetrics, by L. B. Bouchelle, M.D., 88. Treatment of Fistula in Ano—A New Method, by I. J. Suggs, M.D., 89. A Case of Exophthalmic Goitre, by John A. Liddell, M.D., 90. Treatment of Catarrh, by W. F. Duncan, M.D., 91. Chloral in Puerperal Eclampsia, by M. W. Warfield, M.D., 94. Metric System, 95. Treatment of Lupus, 96.

ABSTRACTS AND GLEANINGS.

Pimply-face Acne; Puerperal Convulsions, 97. Sending Consumptives to Travel; Phimosis a Cause of Rupture in Children; Anorexia; Milk Diet in Cystitis, 98. Notes from Practice, 99. Prognosis in Cerebral Hemorrhage; Enuresis Nocturna; Oil of Aleurites Triloba as a Cathartic, 100. Picrate of Ammonia; Benzoate of Lithia; How to Cough, 101. Carbolic Acid Spray; Hypodermic Injection in Hernia; Baldness, 102. Treatment of Morbus Coxarius; Tape-worm; Cerebral Apoplexy, 103. Electric Baths; Sulpho-Thymate of Quinia, Distemper; Onychia Scrofulosa, 104. Anteflexion of the Womb; Self-generating Disinfectant; Treatment of Albuminuria; Cracked Nipples; Insufflation Powders vs. Nasal Douche, 105. Viburnum Prunifolium; Liquor Bismuthi; Nitrite of Amyl; Post-partum Hemorrhage; Injecting a Tumor, 106. Simple Treatment of Quinsy; Gleet; Neurosis of the Heart; Pregnancy; To Prevent Boils, 107. Wounds; Blepharitis; Hooping cough; Obstetric Forceps; Scarlet Fever, 108. Acetate Potass.; Uric Acid Diathesis; Chronic Bright's Disease; Oleate of Zinc, 109. Treatment of Shock; Chloroform Narcosis; Icteric Urine; Milk Diet; Tetanus, 110.

SCIENTIFIC ITEMS.

A Stoppage of the Falls of Niagara; Where the Sun Jumps a Day; Constitution of Matter in the Gaseous State, 111. Sanitary Uses of Gunpowder; The Struggle for Existence; Influence of Sun Spots; Wonders of the Atmosphere; Human Population, 112.

PRACTICAL NOTES AND FORMULÆ.

Pruritus Vulvæ; Vaginismus; Lotion in Pityriasis of the Face, 113. Spirit of Walnut for Vomiting; Asclepias in Scrofula; Bronchitis; For Pleurodynia, 114. Gonorrhœa; Ergotin Suppositories; Chapped Hands; For Impotency; Laxative and Anodyne Pill, 115. Catarrhal Fever; Laxative Mixture; Vaginitis; Grisolle's Pills, 116.

EDITORIAL AND MISCELLANEOUS.

State Med. Association; American Med. Association; Catarrhal Fever, 117. The Fancher Case; The Yellow Fever Commissions; Obituaries, 118. Book Notices, 119. Give us Credit; Special Notices, 120.

